# TCR Signaling in T Cell Memory

**DOI:** 10.3389/fimmu.2015.00617

**Published:** 2015-12-10

**Authors:** Mark A. Daniels, Emma Teixeiro

**Affiliations:** ^1^Department of Molecular Microbiology and Immunology, School of Medicine, University of Missouri, Columbia, MO, USA

**Keywords:** T cell receptor, T cell, protective immunity, immune memory, signaling

## Abstract

T cell memory plays a critical role in our protection against pathogens and tumors. The antigen and its interaction with the T cell receptor (TCR) is one of the initiating elements that shape T cell memory together with inflammation and costimulation. Over the last decade, several transcription factors and signaling pathways that support memory programing have been identified. However, how TCR signals regulate them is still poorly understood. Recent studies have shown that the biochemical rules that govern T cell memory, strikingly, change depending on the TCR signal strength. Furthermore, TCR signal strength regulates the input of cytokine signaling, including pro-inflammatory cytokines. These highlight how tailoring antigenic signals can improve immune therapeutics. In this review, we focus on how TCR signaling regulates T cell memory and how the quantity and quality of TCR–peptide–MHC interactions impact the multiple fates a T cell can adopt in the memory pool.

## Introduction

Upon infection or vaccination, T cell receptors (TCRs) recognize antigen bound to MHC molecules on the surface of antigen-presenting cells (APCs). Antigen recognition results in the transduction of TCR signals that enable T cell differentiation. In this process, TCR signals cooperate with cytokine, costimulatory, chemokine, integrin, and metabolic signals to regulate the acquisition of effector function and the generation of T cells with very different phenotypes. The degree of synergy between all the signals mentioned above leads to changes in the levels of a set of transcription factors that ultimately will determine distinct T cell fates. These include CD8 short-lived effectors, CD4 T helper: Th1, Th2, follicular helper T cell (Tfh), GC (germinal center)–Tfh, T cell effector-memory cells (T_EM_), T cell central-memory cells (T_CM_), T cell resident memory cells (T_RM_), T memory stem cell, or lymphopenia-induced proliferation (LIP)-memory T cell, etc.

Although a great body of work supports the contribution of the inflammatory and local tissue environments to T cell differentiation and memory, emerging data suggest that TCR signaling itself is crucial in this process, especially for T cell memory development. In this review, we discuss these data and propose the idea that TCR signaling is an essential component that enables the integration of environmental cues that shape the T cell memory pool.

## TCR Signal Quantity, Quality, and T Cell Fate

T cell receptor signals are conditioned by different biophysical and biochemical parameters. The affinity of the TCRαβ heterorodimer for antigenic peptide–MHC molecules (pMHC), the dose of antigen presented on the surface of APCs, and the duration of the TCR–pMHC interaction, all determine the strength of TCR signals (1, 2). For a long time, the prevalent idea has been that memory development requires an intermediate to high overall signal strength. Signals that were too weak resulted in only a few memory cells that were not fit to survive or respond. By contrast, signals that were too strong led to terminally differentiated effectors (3). This idea holds true on studies focused on T cell clones that respond to immunodominant epitopes or that bind with high affinity to cognate antigens. In these studies, these high-affinity T cells are recruited in large frequencies to the memory pool, in part due to their greater expansion (4–8). However, in the context of infection or lymphopenia, even very low affinity antigens support memory development (9–13). These data suggest that TCR affinity alone is not predictive of memory outcome. Indeed, other studies have found the duration or *t*1/2 of the pMHC–TCR interaction (both equilibrium and aggregate half-life) can serve as a better predictor of memory fate (14, 15). Thus, recent work has shown that Th1 memory fate correlates with long TCR–pMHC *t*1/2 times and not with the affinity of the pMHC–TCR interaction or the ability to expand (16). The relationship between TCR–pMHC I dwell time and CD8 T cell memory commitment is currently less clear. It is most likely distinct from CD4 T cells, especially when considering the different contributions of CD4 and CD8 co-receptors to the stability of the TCR–pMHC interaction and the narrower range of effector functions for CD8 T cells (17, 18). The timing and availability of the antigen is also important for making the memory “choice.” One report has suggested that early-sustained T cell–APC interactions were absolutely required for the response of memory cells (but dispensable for the acquisition of effector function), although a clear effect in memory generation was not provided (19). By contrast, other reports have shown that shortening TCR stimulation early or late in the response favors the generation of memory T cells (20–23). Collectively, these studies illustrate that a strict quantitative model of TCR signaling cannot easily explain T cell memory commitment/function and suggest that the efficiency of an antigen to assemble the TCR signals that specifically support memory may be more complex than originally anticipated.

An alternative to the quantitative model of TCR signaling for memory is a model that considers the quality of TCR signaling. In other words, the TCR signals that support the development of memory may be qualitatively different from the ones required for other T cell functions (effector function or proliferation). TCR signal quality could be determined by the strength of the TCR signal. In this line, Jenkins and colleagues recently showed how differences in antigen dose/aggregate p-MHCII dwell time can lead to different CD4 T cell lineage choices. They posit a model where a low amount of TCR signaling supports Tfh development, an intermediate amount induces Th1, and large amounts of TCR signaling enables GC–Tfh differentiation (15, 24). For CD8 T cells, a decreasing potential model has been proposed where the weaker the signal a T cell receives (over a certain threshold), the higher the likelihood of the cell to enter in the memory pool (25, 26). This model does not distinguish between antigenic or inflammatory signals nor does it take into account the input of signals from the local tissue environment in determining the phenotypic diversity of the memory pool. However, it is consistent with the fact that very weak TCR signals (even in the range of self) are sufficient to support the memory program (10, 12).

In light of this, it is possible that different T cell outcomes are achieved at different TCR signaling thresholds. This is not a novel concept [reviewed in Ref. (27, 28)]. Seminal work by Valittutti and Lanzavechia originally determined that proliferation required higher antigen doses than IFNγ secretion or cytotoxicity in human T cell clones (29). *In vivo*, it has also been reported that CD8 T cell proliferation requires longer and higher affinity TCR–pMHC interactions than what is required for the acquisition of effector function or memory differentiation (10, 30). Thus, memory fate may be supported by low-grade TCR signals, which are sufficient for the acquisition of memory programing but are not strong or continuous enough to “burn the differentiating T cell to death.” Consistent with this, T cells favor the expression of memory-associated transcription factors (23) and preferentially develop into protective memory T cells in the context of very weak TCR signals (10, 13, 23). These, together with the fact that memory precursors can be detected early in the immune response raises the hypothesis that memory development may be a default pathway whose TCR signaling threshold is lower than the threshold required for full expansion or to become short-lived effector (26, 31). T cells survival capacity may be established depending on the signals that the individual T cell clones continue to experience during the course of an immune response. That is, T cells exposed to strong or continued signals become short-lived effectors destined to die (32), whereas T cells receiving slightly weaker signals are directed into the T_EM_ phenotype. This fosters the intriguing idea that very early in the response a set of T cells are directed down the memory path, perhaps, to ensure diversity within the memory pool (33, 34).

Given that T cells can give rise to a battery of daughter cells of very different phenotypes and fates; most likely, T cells are not predetermined to acquire a specific outcome at the naïve level (35–37). Therefore, qualitatively different biochemical input must determine each of the fates a T cell can adopt during an immune response. These qualitatively different biochemical signals may be defined early in the response or progressively, by a combination of factors that act at the same time, or in a multistage process. It is clear that inflammation and tissue-specific signals shape the phenotypic determination of T effector and memory cells; yet, experimental evidence still indicates that TCR signals, by themselves, are key at inducing memory programs and enabling the T cells ability to receive the extrinsic signals that help to determine the diversity of T cell fates (1, 23, 24, 38). In this scenario, TCR signal quality may be defined independently of the quantity of the TCR signal and dictated by the singular ability of the antigen to efficiently assemble a “signalosome” for memory fate that is distinct from the one required for other T cell functions (discussed in detail below). This is supported by studies where a point mutation in one of the constant domains of the TCR allows for expansion and acquisition of effector function but severely impairs CD8 memory development (39). In this model, the properties of the TCR–pMHC interactions are intact, affinity and dwell time are not compromised. Furthermore, there is not an overall reduction in TCR signaling but rather a defect only in the activation of TCR-dependent NFκB signaling (39). Thus, TCR signaling to memory may be defined by the ability of the antigen to induce the activation of specific signaling pathways key to launch the memory program. In agreement with this, specific inhibition of other signaling pathways, such as Wnt, mTOR, or NFκB, affects T cell memory fate decisions very differently (39–41).

## TCR Signal Strength Impacts TCR Signal Quality

At the risk of being confusing, it is still important to note that the composition of the “memory TCR signalosome” may also change depending on the TCR signal strength. Thus, while the transcriptional signatures that describe the different “flavors” of memory cells may be unique, there may be multiple intracellular signaling options that lead to each fate. Evidence for this has been revealed in studies where T cells defective in the TCR signalosome that supports memory in the context of strong TCR signaling, strikingly were able to regain the ability to differentiate into memory upon challenge with weak antigens (42). In this case, memory development correlated with the capacity to activate NFκB signals and happened even when the activation of other signaling pathways was impaired (42). This suggests that weak and strong TCR ligands induce distinct signalosomes rather than different levels of the same signalosome. Similar conclusions can be reached from the studies of Tubo and Jenkins where depending on the TCR signal strength/TCR–pMHC dwell time/antigen dose, distinct transcriptional profiles could be achieved that supported Tfh, Th1, and GC–Tfh (15, 24). Whether the transcriptional profiles that lead to distinct lineages are governed by the early induction of different TCR signalosomes or controlled by different negative feedback circuits at the level of the nuclei or both remains to be determined.

**Figure 1:**
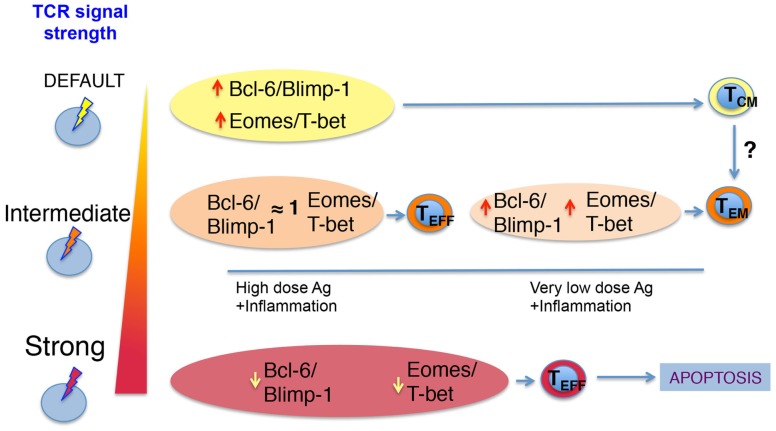
**Proposed model of how TCR signal strength regulates CD8 T cell memory differentiation**. The interactions of TCR–pMHC on a naïve CD8 T cell triggers TCR signals of varying strength. A default TCR signal leads to low levels of T-bet and Blimp-1 and higher levels of Eomes and Bcl-6 skew the naïve T cell into a central-memory phenotype. This weak TCR signal can be achieved by strong or very weak ligands. If the TCR signal is intermediate, then T cells integrate IL-2 and other pro-inflammatory signals, which allow for similar up-regulation of all transcription factors depicted. This ensures the acquisition of effector function early in the response. Once antigen and inflammation decrease due to clearance of the pathogen, effector T cells decrease their levels of Blimp-1 and T-bet and regain higher ratios of Bcl-6/Blimp-1 and Eomes/T-bet allowing them to become memory T cells. For the cases where the naïve T cell receives very strong TCR signals, the Bcl-6/Blimp-1 and Eomes/T-bet ratios are too low and ultimately lead to apoptosis.

How can different TCR signal strengths imprint distinct biochemical signatures that lead to different T cell fates? A model for CD4 T helper differentiation has been proposed where the TCR can support two types of signals (24). One is induced by default and commits the cells into a lineage. This may be the case of the signal that supports Bcl-6 expression in Tfh. The other signal is proportional to the strength of the TCR and implies the expression of other transcription factors, such as Blimp-1 and T-bet for Th1 (43, 44). The higher the dose of antigen, the higher the proliferation and the generation of the progeny of the originally committed Th1 clones. When the TCR signal is too strong, however, Blimp-1 levels are induced to high levels leading to apoptosis of Th1 clones (45, 46). On the other hand, the Tfh clones that originally committed to this lineage, proliferate, induce higher levels of Bcl-6, and eventually differentiate into GC–Tfh (24). A similar model could be applied to CD8 T cells, where a default signal triggered by any engaged TCR, even in the context of very weak TCR ligands, could induce the expression of memory-associated factors Bcl-6 and Eomes (but low levels of Blimp-1 and T-bet) (23). In these conditions, weak TCR ligands would favor the generation of memory T cells with a T_CM_ phenotype over the generation of effector CD8 cells. If T cells encounter stronger TCR signals, then the ratio of Bcl-6/Eomes to Blimp-1/T-bet transcription factors would change. Now, T-bet and Blimp-1 expression would increase, supporting the generation of a large number of effector cells. When the antigenic signal is too strong, T-bet and Blimp-1 levels would increase and Bcl-6 and Eomes levels decrease. This would force the T cells that took the effector path to continue dividing and die (23, 44). By contrast, T cells that received an intermediate TCR signal would be skewed into memory due to a decrease in their T-bet/Blimp-1 levels and/or a natural recovery of the default signal that keeps high levels of Eomes and Bcl-6. Therefore, the TCR signal strength a T cell or their progeny receives may dictate its longevity while specific phenotypes may be further shaped by a continuum of antigenic signals, inflammatory cytokines, and local environmental factors encountered in the course of the response. In agreement with this, very low affinity TCR ligands induce a signal that supports high Eomes/T-bet ratios and high levels of Bcl6 that favor memory development. By contrast, high-affinity TCR ligands at the same dose support low Eomes/T-bet ratios (23) (Figure 1).

This model reconciles the idea of “one cell-multiple fates” with the current models of memory ontogeny. However, it is difficult to test in T cell population studies that cannot track the antigenic experience of individual cells or distinguish between the different precursors of each T cell fate. One question, though, remains to be explained in this model. This is what keeps a T cell in the naïve stage versus the T cell that develops into memory (T_CM_) under very weak TCR signaling. The answer to this question may lie in the dose of the weak antigen and the cytokine milieu. For example, T cells exposed to high doses of weak antigens (in lymphopenic conditions) and cytokines IL-7 and IL-15 differentiate into cells of a T_CM_ memory phenotype (13). Alternatively, the ability of a T cell to remain naïve or differentiate into a T_CM_ phenotype may depend on the TCR signals that it received during thymic development. A hint of how this process may be modulated comes from studies monitoring CD5^hi^ and CD5^lo^ T cell populations in the periphery. Expression of CD5, a negative regulator of TCR signals (47, 48), directly correlates with the strength of the signal generated by the selecting self-pMHC ligand encountered in the thymus. Despite this, peripheral CD5^hi^ T cells exhibit a pre-activated profile that after TCR stimulation leads to stronger responses (49–51). It is possible that the expression of CD5 conditions whether a naïve T cell responding to foreign antigen will reach a TCR signaling threshold that will direct it into one T cell fate or another. Although this has not been formally tested, these studies point to the idea that a T cell’s TCR signal history can greatly influence its fate as the environment around it changes (inflammation, tissue distribution, etc.).

## How TCR Signaling Regulates Memory Development

In recent years, the field has focused a great deal of attention on inflammation and other extrinsic factors and has relegated TCR/antigenic signals to have a minimal role in the process that establishes the clonal heterogeneity of the T cell effector or memory pool. Recent work has brought the TCR back to the forefront of this debate. We have generated a mouse model where T cells bearing a point mutation in the transmembrane domain of the TCRβ chain (βTMDmut) exhibit partial TCR signaling but no other alterations in peptide–MHC–TCR recognition or cytokine signaling. Upon *Listeria* infection, βTMDmut cells were severely defective in generating memory T cells and memory responses (39). This was despite the normal ability of the βTMDmut cells to proliferate and differentiate into effector T cells. Interestingly, the defect in TCR signaling to memory led to a failure to generate “IL-7R^hi^-memory precursors,” and it was not due to impairment in receiving inflammatory or homeostatic input (42). These results challenged the idea that the TCR is a mere spark plug in the T cell memory differentiation process and showed that the TCR signaling requirements are not the same for all T cell outcomes. This idea is further supported by a study from Smith-Garvin and colleagues, which also explored the role of TCR signaling in CD8 T cell differentiation by targeting a downstream intermediate of the TCR signalosome, SLP-76. Employing a knock-in mouse model that express mutant SLP-76, they showed compelling evidence that the TCR signaling requirements for CD8 effector and memory development are different (52). Furthermore, another study using a conditional SLP-76 model also suggested that tonic TCR signals are required beyond the peak of the response to maintain memory CD4 T cell homeostasis and to regulate CD8 memory generation (53, 54). Noteworthy, these and other recent studies (52, 55, 56) have also helped to consolidate the idea that not all the fates that a T cell can adopt are interrelated; that the potential of a T cell to choose the path toward a specific fate and not another can already be determined early in the response; and finally, that not only extrinsic factors but also cell intrinsic TCR-dependent signals or programs may be important to establish the heterogeneity of the effector and memory pools.

How TCR signaling to memory is defined biochemically at the level of signal transduction, transcriptional regulation, and metabolism is beginning to come to light. TCR stimulation results in the activation of several signaling pathways [such as Ca^2+/^NFAT; CBM/PKCθ/NFκB, Vav/Rac/POSH/JNK, RasGRP, or Sos-Ras/RafK/ERK, PI3K, mTOR, Wnt (57, 58)] that, in some cases, are shared with other surface receptors. This is the case for CD28, chemokine receptors, or some TNF receptors that can each utilize membrane proximal intermediates of TCR signaling, such as PI3K or PKCθ (59–61). To date, attempts to demonstrate the role of these specific pathways in T cell differentiation have implied the use of gain/loss of function approaches based on the overexpression of dead or constitutive active forms or a complete deletion of intermediates of the signaling cascades under study. It is important to keep in mind that these approaches can lead to an imbalance in the signaling cross-talk that naturally occurs in a T cell under physiological conditions. Thus, while the conclusions of these studies are extremely informative regarding the role of the specific signaling intermediates in T cell differentiation, they cannot be exclusively ascribed to the TCR. We generated a TCR transgenic model where T cells bearing TCRs mutant in the βTMD are specifically deficient in memory differentiation. This model allowed us to connect TCR with signal transduction to memory. We found that the memory defect was not a consequence of an overall change in the activation of the signaling pathways supported by the TCR signalosome. By contrast, memory differentiation-defective T cells were singularly impaired in the induction of the NFκB signaling pathway (39). Furthermore, once βTMDmut T cells regained the ability to induce NFκB signals, their capacity to differentiate into memory T cells was restored (23). Together, these strongly suggest that TCR-dependent NFκB signaling is crucial for the generation of memory T cells.

T cell receptor-dependent NFκB signaling involves the activation of PKCθ, which enables the assembly of the Carma1/Bcl10/Malt1 (CBM) complex. The CBM complex, in turn, recruits the IKK complex (IKKα, IKKβ, and NEMO) to the membrane to be activated in a TRAF2/6 and TAK1-dependent manner (62). The importance of NFκB signaling in memory development is manifested in EDA-ID (anhidrotic ectodermal dysplasia with immunodeficiency) patients, which carry a gain-of-function mutation of IκBα (an NFκB inhibitor) that leads to impaired NFκB activation and a lack of memory T cells (63). Previous reports utilizing murine models targeting IKKβ or IκBα in T cells also indicated that NFκB signaling pathway was important for generation of memory-phenotype T cells (64, 65). More recently, other studies have also involved members of the non-canonical NFκB cascade in the development of memory T cells (66, 67). However, none of these studies revealed the biochemical mechanism behind the role of NFκB in T cell memory, and only the study by Parker and colleagues addressed its role in the context of infection (67).

An important aspect to consider regarding the NFκB pathway is its ability to regulate different T cell outcomes depending on the level of activation, the kinetics of activation, and the developmental stage or type of T cell. For example, deletion of IKKβ results in a defect in generating memory-phenotype T cells. By contrast, chronic activation of NFκB signaling in T cells responding to *Listeria* infection results in increased apoptosis, which results in defective memory (68). This may be related to the fact that IKKβ, while essential for the induction of NFκB, is also involved in the negative regulation of the cascade and can inhibit early TCR signaling (68, 69). In addition, NFκB signals exhibit different waves of induction after stimuli have gone. How this contributes to T cell fate decisions is also not known (70–73). Finally, it is worthy to note that members of the TCR-dependent NFκB signaling cascade differ in their individual contributions to the final T cell outcome in CD8 versus CD4 T cells. Thus, constitutive activation of IKKβ leads to enhanced CD4 negative selection but has no effect on CD8 T cells (74). T-cell-specific deletion of NEMO or TAK1 prevents the development of both CD4 and CD8 peripheral T cells (65, 75). However, PKCθ, Carma-1, or Bcl10 deletion do not appear to contribute to selection in the thymus, but are remarkably involved in CD4 regulatory T cell development. They also regulate Th17 and Th2, but not Th1 differentiation and contribute to the generation of CD4 memory-phenotype T cells (60, 76–78). Murine and human T cells lacking Carma-1 or Bcl-10 are inefficient at developing a CD8 and CD4 memory-phenotype T cells (79, 80), although the role of these intermediates in T cell memory development has not been fully characterized in the context of infection. An important role for TRAF-6 in CD8 T cell memory but not effector development has also been described (55). TRAF-6 contributes to IKKγ activation and NFκB induction and we have noticed that CD4 TRAF-6 deficient T cells exhibit defects in IκBα phosphorylation and degradation, together with the described enhanced phosphorylation of PI3K/Akt (81). Interestingly, the role of TRAF-6 in CD8 T cell memory development is mediated by the regulation of fatty acid metabolism, although whether this occurs in an NFκB-independent fashion is unclear (55). Collectively, these studies show that NFκB-dependent T cell outcomes are determined by multiple and complex mechanisms. Thus, future research will need to consider all of the aspects described above to thoroughly understand how this signaling pathway regulates T cell effector responses and memory development.

Ras/ERK, PI3K/Akt, and mTOR signaling pathways are also induced upon TCR stimulation and have recently been linked to the development of memory T cells. Ras is a GTPase that is activated by Sos or RasGRP, both GEFs are actively regulated by the TCR signalosome (57). The isoform N-Ras is crucial for ERK-independent regulation of CD8 T cell memory development via regulation of Eomes (82, 83). On the other hand, mTOR signaling was originally described as a key regulator of pro-inflammatory IL-12 and homeostatic IL-7 cytokine signals in CD8 memory programing, particularly through its control of the transcription factors T-bet and Eomes (41, 84). Additionally, mTOR is a central regulator of cell metabolism, growth, survival, and proliferation. mTORC1 (the rapamycin target) is activated upon TCR stimulation (85) via Carma-1 and Malt-1 (86); however, its continuous activation depends on the integration of TCR signals with other cytokine signals, such as IL-12 (41). mTORC2 plays a more important role in cell survival and cytoskeletal regulation (87) and can activate Akt to control mTORC1 (88). The relative contribution of mTORC1 and mTORC2 in T cell memory development has remained elusive, although a recent study by Powell and colleagues supports a differential role for these complexes in CD8 T cell effector/memory responses and memory maintenance (89). How the two mTOR complexes interpret differences in TCR signal strength in the context of other environmental cues to direct the metabolic changes that accompany the transition to memory is unclear. Remarkably, agonistic TCR stimulation results in strong activation of ERK and PI3K/Akt, both involved in the activation of high levels of mTORC1. This supports the idea that strong TCR signals are optimal for effector differentiation and may be detrimental for programing memory longevity via transcription factors such as IRF-4, Blimp-1, or T-bet that can suppress Eomes expression (90, 91). Little is known of how T cells switch from mTORC1- to mTORC2-dependent metabolism to transition from effector to memory. Similarly, it is unclear whether TCR signals regulate mTORC2 and their ability to control the metabolic reprograming that T cells need to differentiate into memory (89). When considering the TCR signal strength constraints of each of the Th subsets (1) with the fact that Th1 and Th17 differentiation is mTORC1 dependent while Th2 is mTORC2 dependent, it is tempting to speculate that mTORC2 might play a major role on memory T cells that have been selected on the basis of low TCR signals, such as T_CM_ (89) or T_RM_, which are highly dependent on Foxo1 → KLF2 → S1P1 [a target of mTORC2 (92, 93)]. On the contrary, T_EM_ cells that are selected in higher TCR signaling conditions might be more dependent on mTORC1.

Another signaling pathway important for memory generation is Wnt signaling. Inhibition of GSK3β mimics Wnt signaling and skews differentiating T cells into a memory stem cell phenotype that provides superior proliferative and antitumor capabilities (40, 94). Wnt signaling requirement for the generation of memory does not appear to require β-catenin (95) but rather targets the transcription factor TCF-1 to regulate Eomes expression (96). TCR signaling can activate Wnt signaling via PI3K/Akt and PKC (40, 97). However, constitutive active Akt signaling diminishes TCF-1, Lef1, and Myc leading to a loss of memory T cells (98). This suggests that, similar to the NFκB pathway, PI3K/Akt signaling appear to have opposing effects on the survival or development of T cell memory depending on the level of activation. This, then, posits the idea that for at least some signaling pathways, there may be specific thresholds that a T cell needs to meet in order to progress into one T cell fate or another. Whether there is a set time in T cell differentiation where the level of the signaling pathway in question fully commits a T cell into a particular fate is not known. It is also possible that at any time intrinsic and extrinsic factors could both contribute to the total level of the specific signaling pathway and determine whether the T cell would progress toward a specific phenotype or another.

Finally, another complex and important issue is signaling cross-talk. Signaling pathways can often synergize and/or regulate each other. This applies to TCR signaling pathways and metabolic signaling pathways. For example, it is well known that NFκB signaling and GSK3β can modulate mTOR signaling (99, 100), and vice versa, mTOR signaling can also modify NFκB signaling (101). Therefore, special attention should be made to the interactions between signaling pathways and how they change depending on the environmental cues, especially when considering therapeutical approaches that aim to activate or suppress a specific signaling pathway.

## How TCR Signaling Shapes the Phenotypic Diversity of Memory

The memory pool is not homogenous and it is now well accepted that different T cell memory subsets, such as T_SCM_, T_CM_, T_EM_, and T_RM_ coexist. Each contributes in unique ways to provide full protection against re-infection. Recent studies are shedding light into how TCR signaling may be regulating each one of these memory fates. TCR-dependent NFκB signals are more important for CD8 T_CM_ cells (23). Wnt signaling, however, appear to be crucial for CD8 T_SCM_ (40) while sustained mTOR signaling is required for the accumulation of T_RM_ in the mucosa (102).

The requirement of TCR signals for T_RM_ is more controversial. Certain features of T_RM_ such as αEβ7 expression may be upregulated in mucosal tissue independent of the presence of antigen. Indeed, cognate antigenic signals are not required for the development of T_RM_ in the intestine, in the female reproductive tract, and in the skin (103, 104). On the other hand, T_RM_ development in lung, CNS, and PNS requires antigen (105). Remarkably, none of these studies has assessed whether tonic self-peptide TCR signaling is required for T_RM_ development or maintenance. Similarly, a fine distinction needs to be made regarding whether there is a TCR signal that favors the generation of T_RM_ upon priming or whether this occurs within the tissue (106).

The relationship between TCR signaling transduction and CD4 T cell memory diversity is also not completely clear. Mounting evidence supports that TCR signal strength plays a role in the generation of T_EM_ versus T_CM_ CD4 cells similar to CD8 T cells, with strong TCR signals guiding the commitment to one of the T_EM_ lineages and weaker stimulation favoring the generation of T_CM_ (107). Recently, T_EM_ (Th1-like) and T_CM_ cell fates have been identified by virtue of their unique expression of T-bet or Bcl-6 (108). It is possible that strong TCR signals driving Th1 differentiation lead to high levels of Blimp-1, which consequently, would repress the expression of Bcl-6, and commit Th1 effectors to the T_EM_ fate (109). Weak TCR signals, in turn, could lead to low levels of Blimp-1 and T-bet and higher expression of Bcl6, supporting T_CM_ differentiation. Alternatively, Bcl-6 could be already expressed as a default and only T cells able to express high levels of IL-2R would repress Bcl-6 while keeping high levels of Blimp-1 and T-bet would direct them into the Th1–T_EM_ phenotype (24, 110). Nevertheless, further research is needed to address how TCR signaling is connected to Bcl6, Blimp-1, or T-bet.

## Maintaining T Cell Memory

CD4 and CD8 memory T cells exhibit distinct requirements for self-peptide–MHC signals to maintain their memory status. Different models have demonstrated that CD8 memory T cell longevity does not require tonic TCR signals (111, 112). On the contrary, self-p-MHC/TCR signals are pivotal for the survival of CD4 memory T cells although a comprehensive description of which type of TCR-dependent signaling cascades are supporting this process is still missing (113, 114). Assessing this would imply the use of conditional systems that allow for specific ablation of the particular signaling pathway at memory. In this line, two studies have addressed the role of early TCR signaling intermediates Lck and SLP-76 at memory using conditional deletion of the genes encoding these proteins. These studies surprisingly show that while Lck is not required for CD8 or CD4 T cell memory (54, 115, 116), SLP-76 is crucial only for CD4 T cell memory homeostatic turnover (53). In studies exploring the role of mTORC1 and NFκB signaling to maintain CD8 memory fate fidelity, we have found that NFκB signals are crucial to promote the longevity and the response of CD8 memory T cells. Interestingly, NFκB at memory does not appear to be maintained by known extrinsic factors but rather is programed early in the response by TCR signals (Knudson and Teixeiro, manuscript in review), unfolding another unexpected role for TCR-dependent regulation in the maintenance of T cell memory.

## The Synergy of TCR and Inflammatory Signals for T Cell Memory

Cytokines in general have been proven to be crucial “transformers” of T cell differentiation covering from full acquisition of effector functions to the generation or maintenance of memory T cells (117, 118). Pro-inflammatory cytokines, in particular, have been suggested to control the sensitivity of the T cell response upon re-exposure to the same antigen (119). The converse axiom whether TCR signals regulate the input or sensitivity of inflammatory signals is also true. The strength of TCR signals can regulate the input of inflammation by directly controlling the expression of pro-inflammatory cytokine receptors and TGFβR (23) on CD8 T cells. The same phenomenon has been reported for CD4 T cells (38) and together, strongly indicates that TCR signals govern sensitivity to inflammation and perhaps to other environmental signals (120) in a hierarchical multistage process.

Antigen and inflammation can, then, regulate each other’s input at different points in the life of a T cell, but the thresholds of this regulation for each of the signals are unknown. Inflammation does not appear to compensate for weak T cell responses of TCR signal strengths that are well above the threshold for thymic selection (121). However, there may be other scenarios where these signals may cooperate or temper each other (122). This could happen at the most proximal membrane level or by regulating similar signaling pathways in the presence or in the absence of cognate antigen. Although this has not been fully explored, a few studies support this hypothesis. For example, it has been described that the chemokine receptor CXCR4, critical for T_CM_ cell renewal and homing to the bone marrow (123, 124), physically associates with the TCR to signal in the absence of foreign antigen (125). This area of research certainly awaits further investigation and it can greatly aid in the identification of molecular checkpoints that can be exploited for therapeutics.

## Concluding Remarks

Memory T cells are an essential part of our immune system that protect against pathogens and tumors. How memory T cells are generated and maintained are still unsolved questions. The memory T cell pool that remains upon infection or vaccination is heterogeneous, containing memory cells phenotypically diverse and with different specialized functions as well. This heterogeneity is the result of how the differentiating T cell integrates antigen, inflammation, costimulation, chemokine, homeostatic, and metabolic signals. At the beginning of the immune response, antigen receptor (TCR signals), pro-inflammatory cytokines (IL-12R/TypeIIFN), and costimulatory signals trigger the differentiation of T cells to acquire effector functions and to become memory cells. A fabulous effort in the field has provided ample insight into how inflammation impacts T cell memory differentiation and the diversity of the memory pool. However, recent data have brought up to stage the role of antigen and TCR signals in determining T cell memory. Experimental evidence supports that TCR signals are not a default but rather an essential component that enables the integration of all environmental cues that shape the T cell effector and memory pool. However, the mechanisms by which the TCR operates to control the final T cell’s outcome are far from clear. As the different layers of regulation of TCR signaling unfold, more questions arise regarding how differences in TCR signal strength regulate the fidelity of the transcriptional program that controls memory development and memory quality or why specific TCR-dependent signaling pathways are more important than others at regulating T cell memory. Likewise, how TCR signaling cooperates in time and space with inflammatory and local tissue environmental signals and what their relative contribution is to memory differentiation is an exciting area that awaits further investigation and is expected to aid greatly in a better design of current immunotherapies.

## Author Contributions

MD wrote and edited the manuscript. ET wrote and edited the manuscript.

## Conflict of Interest Statement

The authors declare that the research was conducted in the absence of any commercial or financial relationships that could be construed as a potential conflict of interest.
